# Correlation between Al grain size, grain boundary grooves and local variations in oxide barrier thickness of Al/AlO_x_/Al tunnel junctions by transmission electron microscopy

**DOI:** 10.1186/s40064-016-2418-8

**Published:** 2016-07-13

**Authors:** Samira Nik, Philip Krantz, Lunjie Zeng, Tine Greibe, Henrik Pettersson, Stefan Gustafsson, Per Delsing, Eva Olsson

**Affiliations:** Department of Applied Physics, Chalmers University of Technology, 41296 Gothenburg, Sweden; Microtechnology and Nanoscience, Chalmers University of Technology, 41296 Gothenburg, Sweden

**Keywords:** TEM, Tunnel junction, Grain size

## Abstract

A thickness variation of only one Ångström makes a significant difference in the current through a tunnel junction due to the exponential thickness dependence of the current. It is thus important to achieve a uniform thickness along the barrier to enhance, for example, the sensitivity and speed of single electron transistors based on the tunnel junctions. Here, we have observed that grooves at Al grain boundaries are associated with a local increase of tunnel barrier thickness. The uniformity of the barrier thickness along the tunnel junction thus increases with increasing Al grain size. We have studied the effect of oxidation time, partial oxygen pressure and also temperature during film growth on the grain size. The implications are that the uniformity improves with higher temperature during film growth.

## Background

The rapid advances of quantum electronics has resulted in an increased need for improved circuit elements (Nakamura et al. 1999; Oh et al. 2006; Vion et al. 2002; Mooij et al. 1999; Kline et al. 2009). During the past decade, more attention has been paid to the undeniable influence of materials and fabrication techniques on the performance of devices, such as superconducting- and normal tunnel junctions (Lang et al. 2004; Tan et al. 2005; Martinis 2009; Roddatis et al. 2011), which are used in a wide variety of devices such as superconducting quantum bits (Clarke and Wilhelm 2008), radiation detectors (Zmuidzinas and Richards 2004), SQUID magnetometers (Clarke 2011), electron pumps (Pothier et al. 1992), and single-electron transistors (Averin and Likharev 1986).

A key point in the fabrication of junction-based devices with controlled and engineered properties, is to have a correct understanding of the microstructure of the different parts of the device. For example, the unwanted quasiparticle tunneling (quasiparticle poisoning) in superconducting Josephson quantum bits (qubits) can be suppressed by engineering the gap profile of the device, which can be done by altering the thickness of superconducting layers (Court et al. 2008; Gunnarsson et al. 2004; Aumentado et al. 2004; Yamamoto et al. 2006). Another important aspect is the fabrication reproducibility of the junctions, which increases the probability of having devices with well defined properties. Understanding the grain size distribution in the thin Al films and the factors that can control the grain size would lead to tailored fabrication of junctions with more homogenous tunnel barriers.

Formation of aluminum oxide on Al film has been studied extensively. The composition and structure of the thin aluminum oxide layer formed by thermal oxidation are found to be sensitive to the oxidation parameters such as oxygen pressure, oxidation time and substrate temperature (Snijders et al. 2002; Cai et al. 2011; Flodström et al. 1976; El-mashri et al. 2006). In addition, there is since long significant attention paid to the aluminum oxide barrier in Nb/AlOx/Al/Nb tunnel junctions where the growth of the oxide layer also takes place an Al film e.g. Kang et al. (2014), Shiota et al. (1992), Imamura and Hasuo (1991, 1992), Kleinsasser et al. (1995, 1996). Thickness of the oxide can also be largely affected by the oxidation conditions as well as the crystalline orientation of the Al film, though there is believed to be a limiting thickness for aluminum oxide directly formed on Al films (Flötotto et al. 2015; Cai et al. 2012; Reichel et al. 2008). Thickness distribution of the oxide barrier in Al based Josephson junctions has been found and characterized directly using microscopy techniques (Zeng et al. 2015; Aref et al. 2014). However, the grain structure of the Al in $\hbox {Al/AlO}_{{\mathrm{x}}}$/Al Josephson junctions, which normally consist of polycrystalline Al as superconducting electrodes, and its effect on the barrier thickness have not been studied previously.

The present work concerns the microstructure of Al layers in $\hbox {Al/AlO}_{{\mathrm{x}}}$/Al tunnel junctions with focus on Al grain size and grain grooves. In addition, the possible effects of different parameters such as oxidation parameters, substrate material and temperature on the grain size of Al and tunnel barrier thickness have been studied. An important correlation between Al grain boundary grooving and a local increase of barrier layer thickness has been identified. This has important implications that can enable more controlled tuning of the junction performance.

## Experiment

Throughout this work, two types of samples were studied. The first type consisted of samples with bi-layer $\hbox {Al/AlO}_{{\mathrm{x}}}$/Al tunnel junctions, whereas the second one consisted of only a single layer of thin Al film. The bi-layer junction samples were fabricated on silicon substrates with a layer of 400 nm thick wet-grown silicon dioxide ($\hbox {Si/SiO}_2$). Two layers of Al were deposited using electron beam evaporation in high vacuum (nominally $10^{-7}$ mbar), with an evaporation rate of 5 Å/s. For these samples, the nominal thickness of the first Al layer was 15 nm, while the top Al layer was 60 nm. In between the two Al depositions, the samples were oxidized using oxidation parameters presented in Table 1.

The samples with a single Al layer were deposited on different substrates using the same electron beam evaporation parameters as for the bi-layer junction samples. These single layers had the thickness of either 15 or 60 nm, in order to be compared with the junction samples. The majority of the samples were fabricated on $\hbox {Si/SiO}_2$ substrates, whereas a few samples were fabricated on Si, silicon with a 400 nm thick silicon nitride layer $\hbox {Si/Si}_3\hbox {N}_4$, or on sapphire $\hbox {Al}_2\hbox {O}_3$. In addition, a few samples were fabricated on Si/SiO$_2$ substrates that were cooled down to $-108\,^{\circ }$C by using liquid nitrogen during evaporation.

From each sample, two types of specimens were prepared; plan-view and cross-sectional transmission electron microscopy (TEM) specimens. Both kinds of specimens were prepared with conventional techniques, including mechanical grinding and polishing with a final thinning step to electron beam transparency using Ar ion milling in a machine called Fischione M1010 (Lilijenfors 2007).

The surface morphologies of the films were characterised using a Zeiss Ultra-55$^{{\mathrm{TM}}}$ scanning electron microscope (SEM). The detailed microstructure of the films was studied using a Titan 80-300 TEM instrument operated at 300 kV and a Tecnai G2 TEM operated at 200 kV. Energy dispersive X-ray (EDX) analysis was used for chemical characterisation in the Titan 80-300 instrument which was equipped with a Super-XTM EDX detector.

**Fig. 1:**
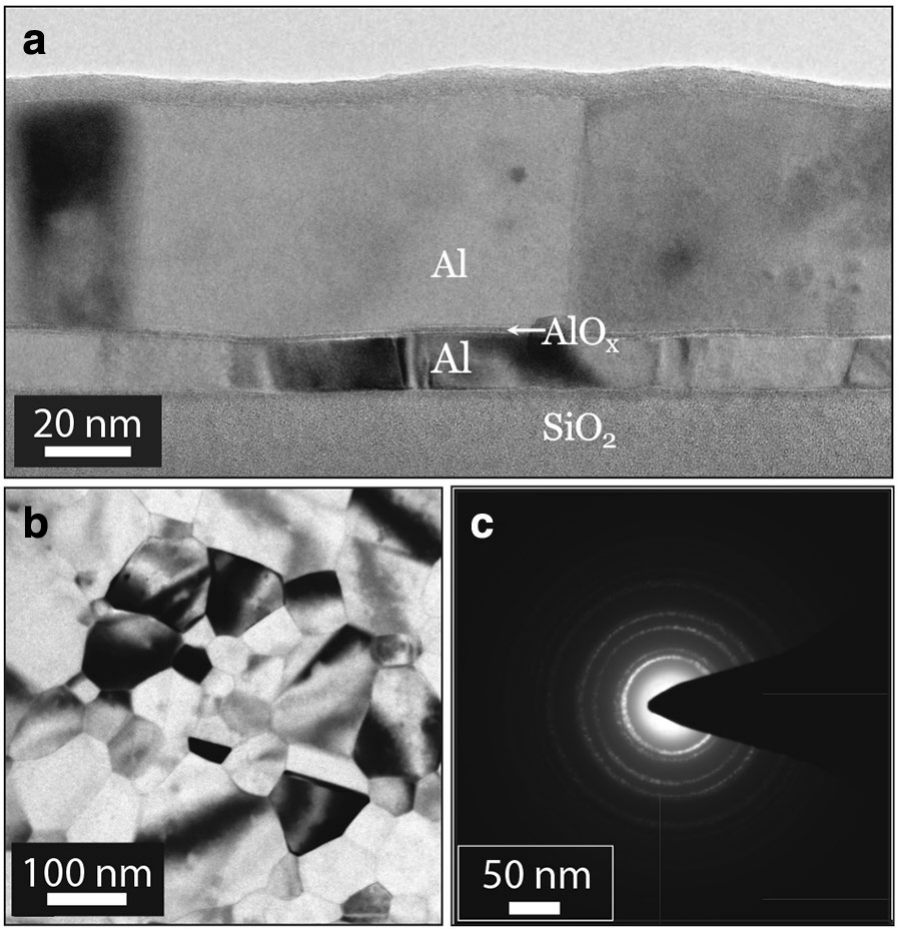
**a** Bright field TEM image of a junction showing the columnar grains in the *bottom* and *top* Al layers of the junction. The polycrystalline nature of these layers is obvious from the diffraction contrast of the image. **b** Plan-view bright field TEM image of a 60 nm-thick Al layer. **c** Diffraction pattern of the layer in (**b**). The polycrystalline nature of the Al layer gives rise to the series of concentric rings

**Table 1:** The oxidation parameters used for sample groups A–D for the bi-layer sample type

Sample group	A	B	C	D
$\hbox {t}_{{\mathrm{ox}}}$ (min)	10	10	30	30
$\hbox {p}_{{\mathrm{ox}}}$ (mbar)	0.01	0.1	0.01	0.1

All devices were oxidized at room temperature in a pure $\hbox {O}_2$-environment

## Results and discussion

### Surface morphology versus grain size

Figure 1 shows a cross-sectional bright field (BF) TEM image of a bi-layer junction, used throughout this work. The two Al layers, separated by a very thin amorphous $\hbox {AlO}_{\mathrm{x}}$ layer, have columnar grains. The diffraction contrast in combination with selected area electron diffraction, Fig. 1b, c, confirms that the bottom and top Al layers are polycrystalline. The Al grains that are darker in contrast are closer to having a crystallographic direction parallel to the incident electron beam. On the other hand, grains that are oriented further away from a crystallographic orientation parallel to the beam diffract less and appear brighter in a BF image.

**Fig. 2:**
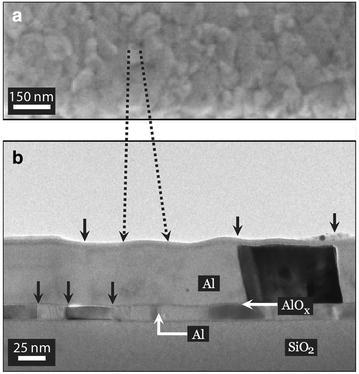
**a** SEM image showing surface morphology of the top Al layers *top-view*. **b** BF TEM image of the junction, the *solid black arrows* indicate the Al grains in the two Al layers. The wavy surface on the *top* Al layer gives rise to the granular contrast observed in SEM as in **a**. It should be noted that the dimension of the surface roughness observed in TEM matches the dimension of the granular structure in the SEM images. This is indicated by the *dashed black lines*

It should be noted that the surface morphology observed by SEM shows a surface topography with a shorter characteristic length scale compared to that of Al the grain size (see Fig. 2). The higher magnification image in the inset shows that the surface of the thin films looks rough and has a granular morphology. These patterns are usually considered as Al grains and therefore Al grain size is often defined by the size of these small features (Court et al. 2008). However, the BF TEM image from the cross-sectional view of the films, in Fig. 2b, shows that these patterns only exhibit the surface morphology of the Al thin films and should not be considered as being representative of the Al grain size.

### Grain size determination

In order to determine the Al grain size of the two Al layers of the $\hbox {Al/AlO}_{{\mathrm{x}}}$/Al junctions, cross-sectional and plan-view specimens from junctions were studied in TEM. In addition, samples with single layers of Al were fabricated on the same substrate with thicknesses similar to the bottom and top layers (15 and 60 nm). In this way the nucleation conditions for both layers were equal and the only remaining variable was the thickness of the layer. As Fig. 1 shows, the Al grains could precisely be observed in the TEM images from both cross-sectional and plan-view specimens. We define the size of the Al grains by measuring their dimension in the plan-view images as well as complimentary information from cross-sectional specimens. This averaged grain size $\langle d \rangle$ was extracted from measuring about 300–500 grains per sample, where the largest dimension of each grain was manually extracted from the TEM images.

Figure 1 shows that the Al films are continous and that the Al grains in both layers have a columnar structure. Moreover, it is evident that there is a significant difference in Al grain size between the bottom and top layers. Our measurements show that in all specimens the average grain size in the top layer $\langle d \rangle _{{\mathrm{t}}} \approx 106$ nm is at least three times larger than the one in the bottom layer $\langle d \rangle _{{\mathrm{b}}} \approx 31$ nm. The reason behind this large size difference is the different thicknesses of these layers, where the bottom layer has a thickness of 15 nm, whereas the top layer is 60 nm thick. This concept is known as the “thickness effect” and will be discussed in detail later on (Mullins 1958).

### Grain growth

#### Normal grain growth, Al grain boundary grooves and local variation in oxide barrier thickness

The plan-view image in Fig. 1b illustrates that the Al grains have a wide distribution of dimensions, ranging from much smaller to much larger than the layer thickness. Measuring the dimension of more than 400 grains in the plan-view images and plotting the grain size distribution of each specimen (Fig. 4), we find that our results are in good agreement with the theories and simulations regarding normal grain growth (Thompson and Carel 1996; Frost et al. 1990, 1992). In other words, our observations concur with the simulated structures where a majority of the grains have a size in the range of up to two or three times larger than the film thickness. In addition, most of the Al grains have five to six nearest grains. Each of the Al films had a columnar structure with only one grain along the direction normal to the substrate interface. The plan-view therefore provide sufficient information about the grain size and also number of nearest neighbour grains. The grain size distributions of our samples follow the log-normal fit (Frost et al. 1990). Consequently, normal grain growth occurred in both, 15 and 60 nm, Al thin films. The driving force for this type of grain growth is lowering of the total free energy of the film, associated with energy reduction of the grain boundary energy (Palmer et al. 1987).

**Fig. 3:**
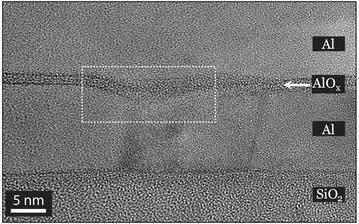
BF TEM image, the *dashed white frame marks* the grain boundary groove that appeared in between two Al grains in the *bottom layer*

According to Mullin’s theory, when a thin metal film is hot enough to allow the atomic migration, a thermal groove will form at the grain boundary. Consequently, such grooving at the layer surface pin the grain boundaries, thus providing an obstacle prohibiting it to propagate (Mullins 1958). The cross section TEM image in Fig. 5 shows a typical groove at a grain boundary between two grains in the bottom Al layer. Here, the grain is growing and a moving grain boundary leaves the groove that it was previously occupying. This appears as a network of lines on the film surface (surface topography) (Mullins 1958). It should be noted that the groove at the Al grain boundary is associated with a local increase in $\hbox {AlO}_{{\mathrm{x}}}$ barrier thickness (Fig. 3).

**Table 2:** Average values $\langle d \rangle$ and standard deviations $\sigma _d$ extracted for the Al grain size distributions for the 15 and 60 nm-thick films in Fig. 4, using the log-normal distribution function

Thickness	60 nm	15 nm
$\langle d \rangle$ (nm)	92 ± 8	38 ± 3
$\sigma _d$ (nm)	45 ± 10	18 ± 4

#### Thickness effect

**Fig. 4:**
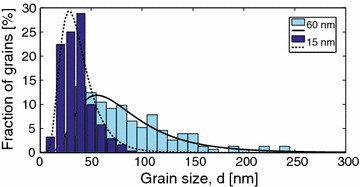
The grain size distributions of the 15 and 60 nm-thick Al films on oxidized silicon. The fitted log-normal distributions (*solid* and *dashed black curves*), with parameters presented in Table 2, follow the common shape for normal grain growth and the tails of the thicker films show the few grains that grew abnormally large

Mullins suggested that the critical situation for a grain boundary to be pinned by a groove or to be released from it is related to the film thickness where thicker films promote boundary motion and thereby yield larger grain sizes (Mullins 1958). This behavior was observed in our samples where the 15 nm-thick Al films have smaller average grain size, and the 60 nm films show a much larger average grain size, see Fig. 4 and Table 2. In both cases, we find that the average grain size is approximately twice the film thickness.

#### Abnormal grain growth

**Fig. 5:**
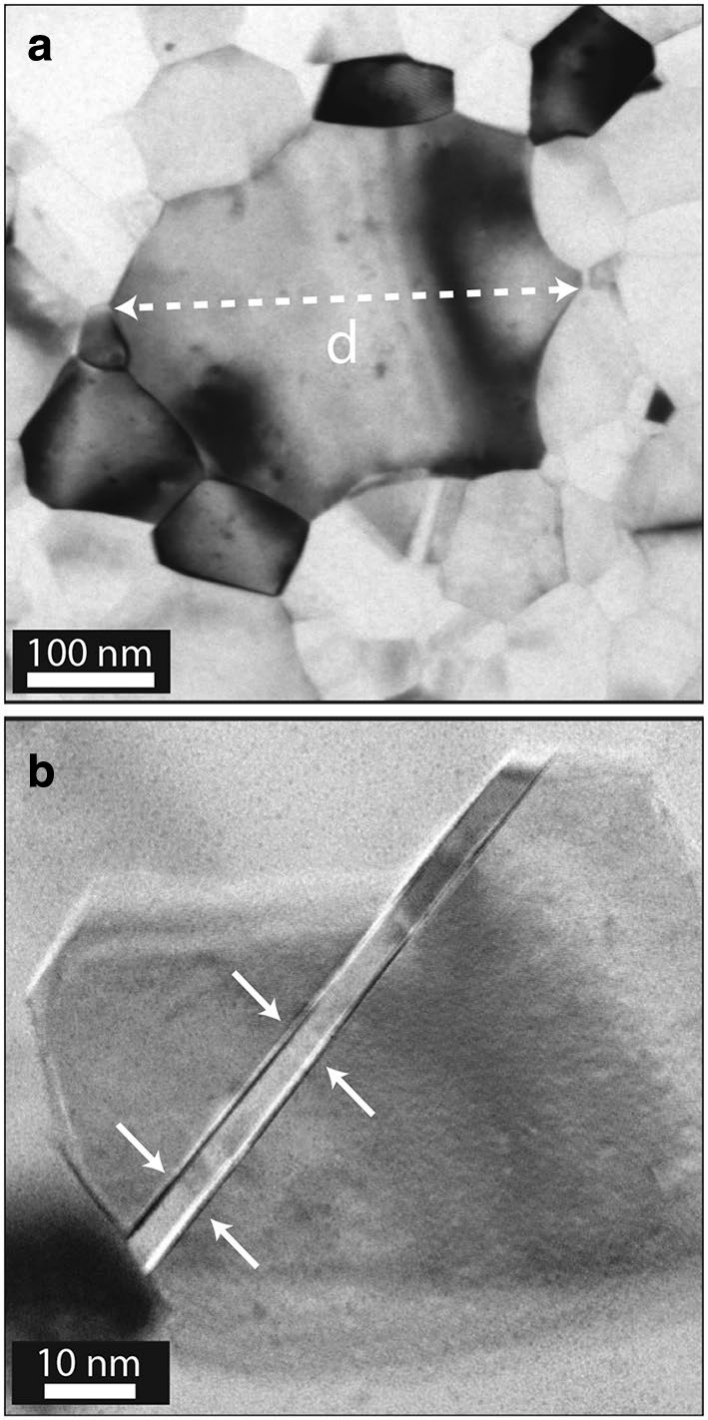
**a** This plan-view BF TEM image shows a grain with abnormal size in a single 60 nm-thick Al film. The largest dimension of this grain is $d = 357\,$nm, as indicated by the *dashed white double arrow*. This size is about six times the film thickness. **b** BF TEM image from another area of the plan view sample showing one Al grain with a twin. The *arrows* indicate the two corresponding twin planes

Figure 5a shows a very large grain surrounded by smaller grains in a 60 nm-thick Al film. The size of this grain is $d \approx 360$ nm, which is almost six times the film thickness. A few of these extremely big grains were observed in all of our specimens (15 and 60 nm thick films) and are seen in the tail of the grain size distribution plots, Fig 4. As we discussed in section a), the normal grain growth stops when the average grain size of the film becomes two or three times the film thickness. The implication of the presence of the abnormally large grains is that some grains continue growing exceeding the normal grain size by another mechanism, *i.e.* abnormal grain growth. According to Mullins, the effect of two grains having unequal free-surface energies and being separated by a grain boundary is that the energy difference acts as a driving force to move the grain boundary towards a configuration with lower total energy (Mullins 1958). Hence, a grain can grow abnormally big if the motion of all its boundaries decrease the total energy (Frost et al. 1992). Both the grain boundary interfaces and the layer surface need to be considered for the interface and Mullins has suggested that grains with different crystallographic orientations may have different free-surface energies. This would then act as a driving force for some grains to grow abnormally big at the expense of other grains in order to minimize the free-surface energy. This raises the question if the abnormally large grains have a certain crystallographic orientation with respect to the substrate surface (Mullins 1958; Frost et al. 1990, 1992; Palmer et al. 1987; Longworth and Thompson 1991). Using selected area electron diffraction we determined the crystallographic orientation of Al grains with abnormally large size and found that they had the [111] direction perpendicular to the substrate, see Fig. 5a. This can be explained by the fact that that this direction is the one with the lowest surface energy in FCC crystals such as Al.

#### Twin boundaries

The area indicated with white arrows in Fig. 5b shows a twin boundary in a 60 nm Al layer evaporated on a Si substrate. Twins are observed in all images of the plan-view specimens and for both film thicknesses. The twin boundaries can be a result of grain growth. Simões, et al., found a linear relationship between the number of twins per grain and the grain size (Simões et al. 2010). It was proposed that twins nucleate at the grain boundaries during the grain growth due to either dissimilar grain boundary mobility or intersection of a mobile grain boundary with a pre-existing twin. On our investigated specimens, twin boundaries occur rather frequently. However, further investigations are needed to give any statistics and this falls outside the scope of the work presented here.

### Effect of oxidation parameters on the grain size

**Fig. 6:**
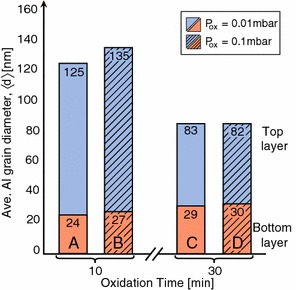
The average (Ave.) grain size dimension $\langle d \rangle$ in the top (*blue*) and bottom (*red*) Al layers for sample groups *A*–*D*. Sample groups *A* and *B* were both oxidized for $\hbox {t}_{\mathrm{ox}} = 10$ min at pressures of $\hbox {p}_{\mathrm{ox}} = 0.01$ and 0.1 mbar, respectively. Sample groups *C* and *D* were oxidized for $\hbox {t}_{\mathrm{ox}} = 30$ min under the oxidation pressures of $\hbox {p}_{\mathrm{ox}} = 0.01$ mbar and $\hbox {p}_{\mathrm{ox}} = 0.1$ mbar, respectively. The data indicates that changing oxidation pressure did not have a prominent effect on the grain size in any of the Al layers. However, the grain size in the top Al layer (tall *blue columns*) is significantly affected by the oxidation time for formation of the $\hbox {AlO}_{{\mathrm{x}}}$ barrier layer, where longer oxidation time results in smaller grains. However, the average grain size in the *bottom layers* (short *red columns*) did not show a significant change with oxidation time

Other parameters to consider, regarding the Al grain size in tunnel junctions, is the oxidation parameters used for forming the $\hbox {AlO}_{\mathrm{x}}$ tunnel barrier. Altering these parameters did not show any noticeable effect on the grain size of the bottom Al layer, which was expected since the oxidation starts after deposition of the bottom layer. However, the grain size in the top Al layer varies with the oxidation time where longer oxidation time results in smaller grains. The Al layers are deposited in a high vacuum chamber at a pressure of $p \approx 3\times 10^{-7}$ mbar, thus the thermal conductivity in the chamber is negligible and the substrate temperature does not change noticeably after deposition of the first Al layer. Nevertheless, when oxygen with a dynamic flow is introduced into the chamber in order to form the tunnel barrier, an additional cooling mechanism is launched to the system. Therefore, longer oxidation time results in a substrate with lower temperature that causes higher density of nucleation centers and hence smaller grain size (Ohring 2002). These results are presented in Table 3 and illustrated in Fig. 6. Changing the oxidation pressure did not show a noticeable effect on the Al grain size.

**Table 3:** The resulting average grain sizes $\langle d \rangle$ for the top and the bottom Al layers for the four different sample groups A–D

Sample group	A	B	C	D
$\hbox {t}_{{\mathrm{ox}}}$ (min)	10	10	30	30
$\hbox {p}_{{\mathrm{ox}}}$ (mbar)	0.01	0.1	0.01	0.1
$\langle d \rangle _{{\mathrm{t}}}$ (nm)	125	135	83	82
$\langle d \rangle _{{\mathrm{b}}}$ (nm)	24	27	29	30

### Effect of substrate material and its temperature

**Fig. 7:**
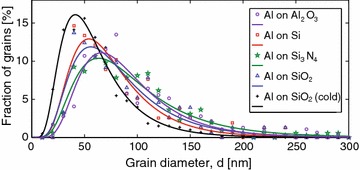
(color online) The log-normal grain size distributions for 60 nm Al deposited on different substrates, with parameters listed in Table 4. The standard deviation of the distribution did not change significantly with substrate material nor did the choice of substrate significantly affect the expectation value of the grain size. However, a lower substrate temperature during growth ($-108\,^{\circ }$C) reduces the grain size substantially as shown by the *black curve*

In order to explore the possible effect of the substrate material on the grain size, the same type of investigations were carried out also on samples with 60 nm Al evaporated on sapphire ($\hbox {Al}_2\hbox {O}_3$), intrinsic silicon (Si), and silicon nitride ($\hbox {Si/Si}_3\hbox {N}_4$). The results are shown in Fig. 7, where the grain size variation follows a log-normal distribution (Frost et al. 1990).

The results indicate that a change of the substrate material does not cause a significant change in the Al grain size. Another important factor that could influence the grain size is the substrate temperature. Therefore, the same study was carried on Al layers grown on substrates with lower temperature. The substrates were cooled down to $-108\,^{\circ }$C by use of liquid nitrogen. The resulting grains have similar shape and structure while their average grain size is about $40\,\%$ smaller compared to layers deposited on substrates at room temperature (Fig. 7). For example, the average grain size for a 60 nm Al film deposited on a cold $\hbox {Si/SiO}_2$ substrate is 62 nm. This result is in a qualitative agreement with the theory suggesting a reduction of nucleation barrier with lowering the temperature and therefore the number of nucleation centers increases as the temperature decreases (Ohring 2002).

**Table 4:** Average values $\langle d \rangle$ and standard deviations $\sigma _d$ extracted for the Al grain size distributions for the different substrates in Fig. 7, using the log-normal distribution function

Substrate	$\hbox {Al}_{2}\hbox {O}_{3}$	Si	$\hbox {Si}_{3}\hbox {N}_{4}$	$\hbox {SiO}_{2}$	$\hbox {SiO}_{2}$ (cold)
$\langle d \rangle$ (nm)	92 ± 8	78 ± 8	98 ± 8	85 ± 9	62 ± 4
$\sigma _d$ (nm)	46 ± 10	40 ± 10	57 ± 11	48 ± 12	34 ± 5

## Conclusion

The local thickness of the tunnel barrier varies along the junction and our studies show that there is a local thickness increase associated with location of the Al grain boundaries. There are thermal grooves at the boundaries. They develop during film growth and have an important effect on the grain boundary mobility and thus the grain growth. The normal grain growth, which is driven by reduction of the energy associated with the grain boundaries, continues until the average grain size of the film reaches 2–3 times the film thickness.

The surface morphology of the grains arises from the grooves remaining from the grain boundaries that were released. The surface morphology observed by SEM is thus not a fair measure of the grain size.

A few very large grains in the films are formed by an abnormal grain growth mechanism, where lowering the free-surface energy is the driving force. The observed twin boundaries appear as a result of grain growth.

The grain size in the top Al grain showed a dependence on the oxidation time used for forming the tunnel barrier and a longer time resulted in smaller grains. On the other hand, the oxidation pressure did not affect the grain size.

Changing the substrate materials did not alter the grain size significantly. However, the substrate temperature did affect the grain size which increased with increasing temperature. Our observation, that the oxide barrier thickness locally increases at Al grain boundary grooves (see Fig. 3), leads to the conclusion that a larger Al grain size favours a more homogenous barrier layer thickness. The substrate temperature can thus be used to tune the homogeneity of the barrier thickness along the tunnel junction.
